# Molecular and Cytogenetic Characterization of Wild *Musa* Species

**DOI:** 10.1371/journal.pone.0134096

**Published:** 2015-08-07

**Authors:** Jana Čížková, Eva Hřibová, Pavla Christelová, Ines Van den Houwe, Markku Häkkinen, Nicolas Roux, Rony Swennen, Jaroslav Doležel

**Affiliations:** 1 Institute of Experimental Botany, Centre of the Region Haná for Biotechnological and Agricultural Research, CZ-78371, Olomouc, Czech Republic; 2 Bioversity International, B-3001, Leuven, Belgium; 3 Finnish Museum of Natural History, University of Helsinki, FI-00014, Helsinki, Finland; 4 Bioversity International, 34397 Montpellier Cedex 5, France; 5 Division of Crop Biotechnics, KU Leuven, B-3001, Leuven, Belgium; 6 International Institute of Tropical Agriculture, Duluti, Arusha, Tanzania; Ben-Gurion University, ISRAEL

## Abstract

The production of bananas is threatened by rapid spreading of various diseases and adverse environmental conditions. The preservation and characterization of banana diversity is essential for the purposes of crop improvement. The world's largest banana germplasm collection maintained at the Bioversity International Transit Centre (ITC) in Belgium is continuously expanded by new accessions of edible cultivars and wild species. Detailed morphological and molecular characterization of the accessions is necessary for efficient management of the collection and utilization of banana diversity. In this work, nuclear DNA content and genomic distribution of 45S and 5S rDNA were examined in 21 diploid accessions recently added to ITC collection, representing both sections of the genus *Musa*. 2C DNA content in the section Musa ranged from 1.217 to 1.315 pg. Species belonging to section Callimusa had 2C DNA contents ranging from 1.390 to 1.772 pg. While the number of 45S rDNA loci was conserved in the section Musa, it was highly variable in Callimusa species. 5S rRNA gene clusters were found on two to eight chromosomes per diploid cell. The accessions were genotyped using a set of 19 microsatellite markers to establish their relationships with the remaining accessions held at ITC. Genetic diversity done by SSR genotyping platform was extended by phylogenetic analysis of ITS region. ITS sequence data supported the clustering obtained by SSR analysis for most of the accessions. High level of nucleotide diversity and presence of more than two types of ITS sequences in eight wild diploids pointed to their origin by hybridization of different genotypes. This study significantly expands the number of wild *Musa* species where nuclear genome size and genomic distribution of rDNA loci is known. SSR genotyping identified *Musa* species that are closely related to the previously characterized accessions and provided data to aid in their classification. Sequence analysis of ITS region provided further information about evolutionary relationships between individual accessions and suggested that some of analyzed accessions were interspecific hybrids and/or backcross progeny.

## Introduction

Bananas and plantains (*Musa* spp.) are one of the most important food crops with the global annual production exceeding 130 Mt (faostat.fao.org). They are grown mainly by smallholder farmers for local consumption and only about 10% of the world's production is exported. Most of the cultivated bananas are parthenocarpic triploid clones (although diploids and tetraploids also occur), which originated from natural intra- and interspecific hybridizations between various subspecies of *M*. *acuminata* (A genome) and *M*. *balbisiana* (B genome) [1] within the genus *Musa*. The triploids are then classified as AAA, AAB and ABB and include a range of varieties of dessert bananas, cooking bananas and plantains, which provide essential nutrition for millions of people in the humid tropics. Minor group of cultivated bananas derived from the hybridization between the A, B and one of the other recognized *Musa* genomes, the S genome and T genome (*M*. *schizocarpa* and *M*. *textilis*, respectively) [2, 3].

The production of bananas is threatened by various diseases, pests and adverse environmental conditions. This imposes the need for varietal testing and crop improvement supported by preservation of characterized banana diversity. The global *Musa* collection is maintained at the Bioversity International Transit Centre (ITC) hosted by the Katholieke Universiteit Leuven in Belgium [4]. The ITC collection was originally established for long term conservation of diploid, triploid and tetraploid edible cultivars, but eventually it started to be expanded by different wild species and subspecies. Currently, the collection contains more than 1,500 accessions in tissue culture. Newly introduced accessions of wild *Musa* species are a significant source of material to study diversity in *Musa* on a broader scale.

In addition to *M*. *acuminata* and *M*. *balbisiana*, the genus *Musa* contains about 70 species. Based on morphology [5] and chromosome number, the genus was divided into sections Eumusa (x = 11), Rhodochlamys (x = 11), Australimusa (x = 10) and Callimusa (x = 9, 10) [6]. More recently, Argent [7] created a separate section Ingentimusa, which contains only a single species *M*. *ingens* (x = 7). This classification has been questioned recently, and various regroupings were suggested [8]. Genotyping with several types of DNA markers confirmed the need for revision of the *Musa* sections [9–12]. Based on these results, Häkkinen [13] combined the Australimusa, Ingentimusa and Callimusa sections into section Callimusa, and sections Eumusa and Rhodochlamys into a newly created section Musa.

The difficulties with the classification of new cultivars and newly discovered wild *Musa* species using morphological descriptors call for characterization of *Musa* accessions using stable and reproducible characters. One of the basic characteristics of a species is its nuclear genome size. In earlier studies, the nuclear genome size was estimated mainly on edible bananas and their wild ancestors. A genome size of 600–650 Mbp was determined in *M*. *acuminata* and 550 Mbp in *M*. *balbisiana*, clearly discriminating both species [14–16]. Bartoš *et al*. [9] extended the knowledge of nuclear DNA content by representatives of former sections Rhodochlamys, Australimusa and Callimusa. Flow cytometric analysis showed that 2C nuclear DNA contents of Eumusa and Rhodochlamys accessions were overlapping, ranging from 1.130 to 1.377 pg in Eumusa and from 1.191 to 1.299 pg in Rhodochlamys. Species belonging to section Australimusa had 2C nuclear DNA content between 1.435 and 1.547 pg. *M*. *beccarii*, the only studied representative of Callimusa section, had the highest 2C nuclear DNA content (1.561 pg). However, only nineteen accessions were included in that study and the knowledge about genome size remained limited, especially in the section Callimusa.

The number and morphology of chromosomes describe a karyotype, another important characteristic of a species. In case of the genus *Musa*, chromosome number determines sectional classification of individual species. More detailed cytogenetic studies are, however, complicated by the small size of chromosomes, their morphological similarity and lack of chromosome-specific landmarks. To date, identification of all chromosomes within the karyotype is not possible. The only sequences cytogenetically mapped to chromosomes of a wider range of *Musa* accessions are ribosomal RNA (rRNA) genes. While the number of 45S rDNA loci was found conserved within individual sections, the number of 5S rDNA loci ranged from 4 to 8 per mitotic metaphase plate and varied within sections and even within different accessions of a single species [9, 16–18]. Similarly to genome size, genomic distribution of rRNA genes was studied mainly in *M*. *acuminata*, *M*. *balbisiana* and some cultivated varieties.

Unlike the methods mentioned above, genotyping using DNA markers provides higher resolution and is suitable for analysis of genetic diversity and establishment of phylogenetic relationships. Different types of molecular markers, such as RAPDs [19], RFLPs [20, 21], AFLPs [22, 23] or DArT markers [24], were used in diversity studies and molecular characterization of *Musa*. In 2011, a standardized microsatellite-based genotyping platform for molecular characterization of *Musa* germplasm was established [13]. Microsatellites (SSRs) are popular molecular markers because of their high abundance in the genome, co-dominant inheritance, extent of allelic diversity, good reproducibility and the potential for high-throughput analysis and automatisation. They proved to be useful in molecular genotyping of many economically important crops [25–27]. The microsatellite genotyping platform of Christelová et al. [13] is based on 19 microsatellite loci [28–30], which are scored using fluorescently labelled primers and high-throughput, high-resolution capillary electrophoresis. The platform enables discrimination between individual species, subspecies and subgroups of *Musa* accessions and is suitable for characterization of unknown accessions. Based on the results of Christelová *et al*. [13], the Musa Genotyping Centre was established at the Institute of Experimental Botany in Olomouc (Czech Republic) [31] under the auspices of Bioversity International. The database of reference SSR profiles they generate is enlarged with every new accession passing through the pipeline and consequently improves the grouping and enhances the probability of identifying the closest relative or an exact match for unknown accessions. Increasing the number of analysed wild species would give more accuracy to the grouping of the genus *Musa*.

When SSR genotyping provides incongruent results, or in case there is a need to support putative hybrid character of particular *Musa* accessions, the ITS sequence analysis can be used. The analysis of internal transcribed spacer region (ITS1-5.8S-ITS2) of nuclear ribosomal DNA provides additional information on genetic diversity and evolutionary relationships among individual accessions. The ITS region has been one of the most widely used markers in phylogenetic studies of flowering plants [32] and has recently been successfully employed in phylogenetic reconstruction in the family Musaceae [10, 11, 33]. It has been shown that ITS locus can be used for verification of genome constitution of inter- and intraspecific banana hybrids [33].

The aim of this work was to characterize a set of 21 *Musa* accessions which were newly introduced into the ITC collection from Helsinki University Botanic garden with a goal to increase the knowledge about the genetic diversity of wild *Musa* species. Most of the *Musa* accessions have been analyzed for the first time. Nuclear genome size, chromosome number and genomic distribution of rDNA were determined in individual accessions to shed light on their genome structure and evolution. Molecular characterization was performed using SSR genotyping platform and was extended by the analysis of ITS sequence region to verify a putative hybrid origin of some accessions. The analysis was also used to support incongruous results of SSR genotyping and to strengthen the previous knowledge on phylogenetic relationships within the Musaceae family.

## Materials and Methods

### Plant Material and Genomic DNA Extraction

*In vitro* rooted plants of 21 *Musa* accessions (S1 Table; basic description of accessions available at [5] were obtained from the International Transit Centre (ITC, Katholieke Universiteit, Leuven, Belgium). The plants were transferred to soil and maintained in a greenhouse. Fresh unopened (cigar) leaves were harvested, their segments lyophilized and maintained at room temperature until use. Genomic DNA of all *Musa* species used for the SSR genotyping and ITS analysis was isolated from lyophilized leaves using the NucleoSpin PlantII kit (Macherey-Nagel GmbH & Co. KG, Düren, Germany) following the manufacturer's recommendations.

### Estimation of Genome Size

Nuclear genome size was estimated according to Bartoš *et al*. [9]. Approximately 50 mg of a young *Musa* leaf and 10 mg of a soybean leaf (*Glycine max* L. cv. Polanka, 2C = 2.5 pg DNA) which served as internal standard [14] were used for sample preparation. Suspensions of cell nuclei were prepared by simultaneous chopping of leaf tissues of *Musa* and *Glycine* in a glass Petri dish containing 500 μl Otto I solution (0.1 M citric acid, 0.5% v/v Tween 20). Crude homogenate was filtered through a 50 μm nylon mesh. Nuclei were then pelleted (300 *g*, 5 min) and resuspended in 300 μl Otto I solution. After 1 hour incubation at room temperature, 900 μl Otto II solution (0.4 M Na_2_HPO_4_) [34] supplemented with 50 μg/ml RNase, 50 μg/ml propidium iodide and 3 μl/ml 2-mercaptoethanol, were added. Samples were analyzed using Partec PAS flow cytometer (Partec GmbH, Münster, Germany) equipped with 488-nm argon laser. At least 5,000 nuclei were analyzed per sample. Three individuals were analyzed in each accession, and each individual was measured three times on three different days. Nuclear DNA content was then calculated from individual measurements following the formula:

$$2\text{C nuclear DNA content}\;\left[\text{pg}\right]=\;2.5\;x\;{\text{G}}_{1}\;\text{peak mean of}\;Musa/{\text{G}}_{1}\;\text{peak mean of}\;Glycine$$

Mean nuclear DNA content (2C) was then calculated for each plant. Genome size (1C value) was then determined considering 1 pg DNA equal to 0.978×10^9^ bp [35].

Statistical analysis was performed using NCSS 97 statistical software (Statistical Solutions Ltd., Cork, Ireland). One-way ANOVA and a Bonferoni's (All-Pairwise) multiple comparison test were used for analysis of variation in nuclear DNA content. The significance level α = 0.01 was used. Spearman's correlation analysis was used to test the relationship between chromosome number and nuclear DNA content of studied accessions.

### SSR Genotyping

The standardized platform for molecular characterization of *Musa* germplasm [12] was used to genotype all 21 *Musa* accessions. The system is based on 19 microsatellite loci that are scored after the PCR with fluorescently labeled primers and capillary electrophoretic separation with internal standard (GeneScan 500 LIZ size standard, Applied Biosystems). The PCR products were multiplexed prior to the separation and loaded onto the automatic 96-capillary ABI 3730xl DNA Analyzer. Electrophoretic separation and signal detection was carried out with default module settings. The resulting data were analyzed and called for alleles using GeneMarker v1.75 (Softgenetics), manually checked and implemented into the marker panels [12]. The SSR profiles of newly analyzed accessions were integrated to the binary table of SSR profiles obtained in the work of Christelová *et al*. [12] and analyzed together. The genetic similarity matrices based on Nei´s genetic distance coefficient [36] were calculated using PowerMarker v3.25 [37] and the unweighted pair-group method with arithmetic mean (UPGMA) [38] was used to assess the relationship between individual genotypes. The results of UPGMA cluster analysis were visualized in a form of a tree using FigTree v1.4.0 [39].

### Analysis of ITS1-5.8S-ITS2 Region

The ITS region was amplified from genomic DNA using PCR with specific primers ITS-L and ITS-4 [40]. PCR reaction mix consisted of 10 ng genomic DNA, 1.5 mM MgCl_2_, 0.2 mM dNTPs, 1 mM primers ITS-L and ITS-4, 1x PCR buffer and 2U/100 of Dynazyme II DNA polymerase (Finnzymes, Espoo, Finland). Amplification was performed using PTC-200 thermal cycler (BIO-RAD, Hercules, CA, USA), with the following conditions: 94°C for 5 min (1 cycle), 94°C for 50 s, 52°C for 50 s, 72°C for 50 s (35 cycles) and 72°C for 10 min (1 cycle), and PCR products were resolved in 1.5% agarose gels.

PCR products were purified using ExoSAP-IT (USB, Cleveland, OH, USA) according to the manufacturer's instructions, cloned into TOPO vector, and transformed into *E*. *coli* electrocompetent cells (Invitrogen Life Technologies, Carlsbad, USA). For each accession, at least 28 cloned PCR products were sequenced. Sequencing was carried out using the BigDye Terminator v3.1 Cycle Sequencing kit (Applied Biosystems, Foster City, USA) according to the manufacturer's instructions and run on ABI 3730xl DNA analyzer (Applied Biosystems, Foster City, USA). Nucleotide sequences were edited using Staden Package [41]. Sequence boundaries of the spacers were assessed and phylogenetic relationships analysis was performed according to Hřibová *et al*. [33]. Sequence diversity was identified using DnaSAM program [42] with 1000 simulations. SplitsTree4 v4.1.11 [43] was used to construct phylograms based on the Jukes-Cantor model. Non-parametric bootstrapping with 1000 pseudoreplicates was performed to assess the nodal support. Phylogenetic trees were drawn and edited using FigTree program [39].

### Chromosome Preparations and Chromosome Counting

Mitotic metaphase spreads were prepared according to Doleželová *et al*. [17]. Actively growing root tips were pre-treated in 0.05% (w/v) 8-hydroxyquinoline for 3 hrs at room temperature and then fixed in 3:1 ethanol: acetic acid overnight. Fixed roots were washed in a solution of 75 mM KCl and 7.5 mM EDTA (pH 4) and meristem tips were digested in a mixture of 2% (w/v) pectinase and 2% (w/v) cellulase in 75 mM KCl and 7.5 mM EDTA (pH 4) for 90 min at 30°C. Protoplast suspension was then filtered through a 150 μm nylon mesh and pelleted. The pellet was resuspended in 75 mM KCl and 7.5 mM EDTA (pH 4) and incubated for 5 min at room temperature. After pelleting, the protoplasts were washed three times with 70% ethanol. Five μl of the suspension were dropped onto a slide and shortly before drying out, 5 μl of 3:1 fixative were added to the drop to induce protoplast bursting. Finally, the slide was rinsed in 100% ethanol and air-dried.

For chromosome counting, the preparations were stained with DAPI (Vectashield Mounting Medium with DAPI; Vector laboratories). Slides were examined with Olympus AX70 fluorescence microscope. Images were captured using a cooled high-resolution black and white camera and processed using MicroImage software (Olympus, Tokyo, Japan). In each plant, two slides were observed, each with at least five metaphase plates.

### Fluorescence *In Situ* Hybridization (FISH)

Probes for 45S rDNA and 5S rDNA were prepared by labeling *Radka*1 DNA clone (45S rDNA) and *Radka*2 DNA clone (5S rDNA) [44] with digoxigenin-11-dUTP or biotin-16-dUTP (Roche Applied Science). Both probes were labeled by PCR using M13 forward and reverse primers (Invitrogen). Hybridization mixture consisting of 50% formamide, 10% dextran sulfate in 1×SSC and 1 μg/ml of each labeled probe was added onto slides and denatured at 80°C for 3 min. The hybridization was carried out at 37°C overnight. The sites of probe hybridization were detected using anti-digoxigenin-FITC (Roche Applied Science) and streptavidin-Cy3 (Vector Laboratories, Burlingame, USA), and the chromosomes were counterstained with DAPI. The slides were examined with Olympus AX70 fluorescence microscope and the images of DAPI, FITC and Cy-3 fluorescence were acquired separately with a cooled high-resolution black and white CCD camera. The camera was interfaced to a PC running the MicroImage software (Olympus, Tokyo, Japan). At least ten complete metaphases were examined for every accession.

## Results

### Estimation of Genome Size

**Fig 1:**
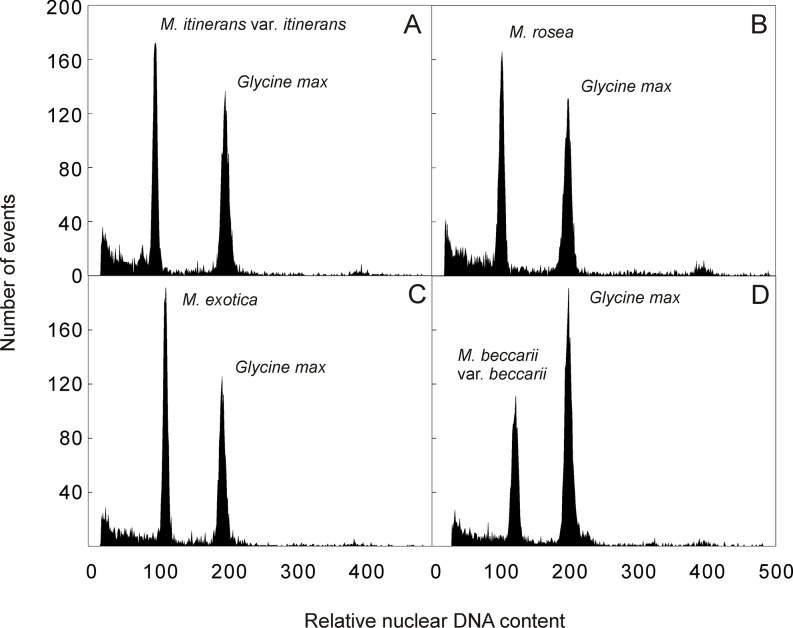
Histograms of relative nuclear DNA content obtained after flow cytometric analysis of propidium iodide-stained nuclei isolated from (A) *M*. *itinerans* var. *itinerans* (ITC.1571) (2C = 1.217 pg); (B) *M*. *rosea* (ITC.1598) (2C = 1.285 pg); (C) *M*. *exotica* (ITC.1532) (2C = 1.442 pg); (D) *M*. *beccarii* var. *beccarii* (ITC.1516) (2C = 1.537 pg). Nuclei isolated from soybean (*Glycine max*, 2C = 2.5 pg) were included as internal reference standard.

The amount of nuclear DNA was estimated after flow cytometric analysis of propidium iodide-stained nuclei. All analyses resulted in histograms of relative DNA content with two dominant peaks corresponding to G_1_ nuclei of *Musa* and *Glycine*, the latter serving as internal reference standard (Fig 1). The 2C nuclear DNA content determined based on the ratio of G_1_ peaks positions ranged from 1.217 to 1.772 pg (Table 1). The differences between the accessions from section Musa were statistically significant with 2C DNA content ranging between 1.217 and 1.315 pg. Much higher interspecific variation (27.5%) of 2C DNA content was observed within the section Callimusa (2C = 1.390–1.772 pg).

**Table 1:** Nuclear DNA content, chromosome number and the number of 45S and 5S rDNA loci.

Section of the genus *Musa*	Accession name	ITC code	2C nuclear DNA content [pg], Mean ± SD	Monoploid genome size [Mbp/1C_x_]	Bonferoni's DNA content grouping*												Chromosome number (2n)	45S rDNA**	5S rDNA**
Musa	*Musa rubinea*	1518	1.310 ± 0.016	641					E								22	2	4
	*Musa x fennicae* (*M*. *siamensis* (male) x *M*. *rosea* (female))	1522	1.261 ± 0.012	617		B	C										22	2	6
	*Musa itinerans* var. *xishuangbannaensis*	1526	1.311 ± 0.017	641					E								22	2	4
	*Musa siamensis*	1534	1.280 ± 0.006	626		B	C										22	2	6
	*Musa itinerans* var. *itinerans*	1571	1.217 ± 0.004	595	A												22	2	4
	*Musa yunnanensis*	1573	1.259 ± 0.011	616		B											22	2	6
	*Musa mannii*	1574	1.282 ± 0.008	627			C										22	2	3
	*Musa laterita*	1575	1.315 ± 0.006	643					E								22	2	6
	*Musa rubra*	1590	1.306 ± 0.01	639				D	E								22	2	6
	*Musa rosea* x *siamensis*	1592	1.279 ± 0.006	625			C										22	2	7
	*Musa rosea* (hybrid)	1598	1.285 ± 0.002	628			C	D									22	2	4
Callimusa	*Musa violascens*	1514	1.428 ± 0.011	698							G	H					20	2	4
	*Musa lutea*	1515	1.432 ± 0.009	700							G	H					20	2	2
	*Musa beccarii* var. *beccarii*	1516	1.537 ± 0.017	752										J			18	6	4
	*Musa campestris* var. *sarawakensis*	1517	1.417 ± 0.008	693							G						20	2	2
	*Musa monticola*	1528	1.390 ± 0.016	680						F							20	2	4
	*Musa beccarii* var. *hottana*	1529	1.673 ± 0.02	818											K		18	6	4
	*Musa borneensis*	1531	1.772 ± 0.006	867												L	20	5	8
	*Musa exotica*	1532	1.442 ± 0.006	705								H					-	-	-
	*Musa campestris* var. *limbangensis*	1535	1.454 ± 0.003	711								H					-	-	-
	*Musa barioensis*	1568	1.480 ± 0.014	724									I				20	2	3

*) Statistical analysis was performed using mean values of 2C nuclear DNA content of individual plants (α = 0.01). DNA content within each Bonferoni's group described by the same letter was not significantly different.

**) Number of FISH signals in a mitotic metaphase plate (2n)

Bonferoni's (All-Pairwise) multiple comparison test revealed twelve groups based on the differences in the nuclear DNA content (Table 1). Six of these groups were represented by only one accession (*M*. *itinerans* var. *itinerans* (ITC.1571), *M*. *monticola* (ITC.1528), *M*. *barioensis* (ITC 1568), *M*. *beccarii* var. *beccarii* (ITC.1516), *M*. *beccarii* var. *hottana* (ITC.1529) and *M*. *borneensis* (ITC.1531)). Four groups comprised different representatives of the section Musa (former sections Eumusa and Rhodochlamys) and two groups included different species belonging to section Callimusa.

**Fig 2:**
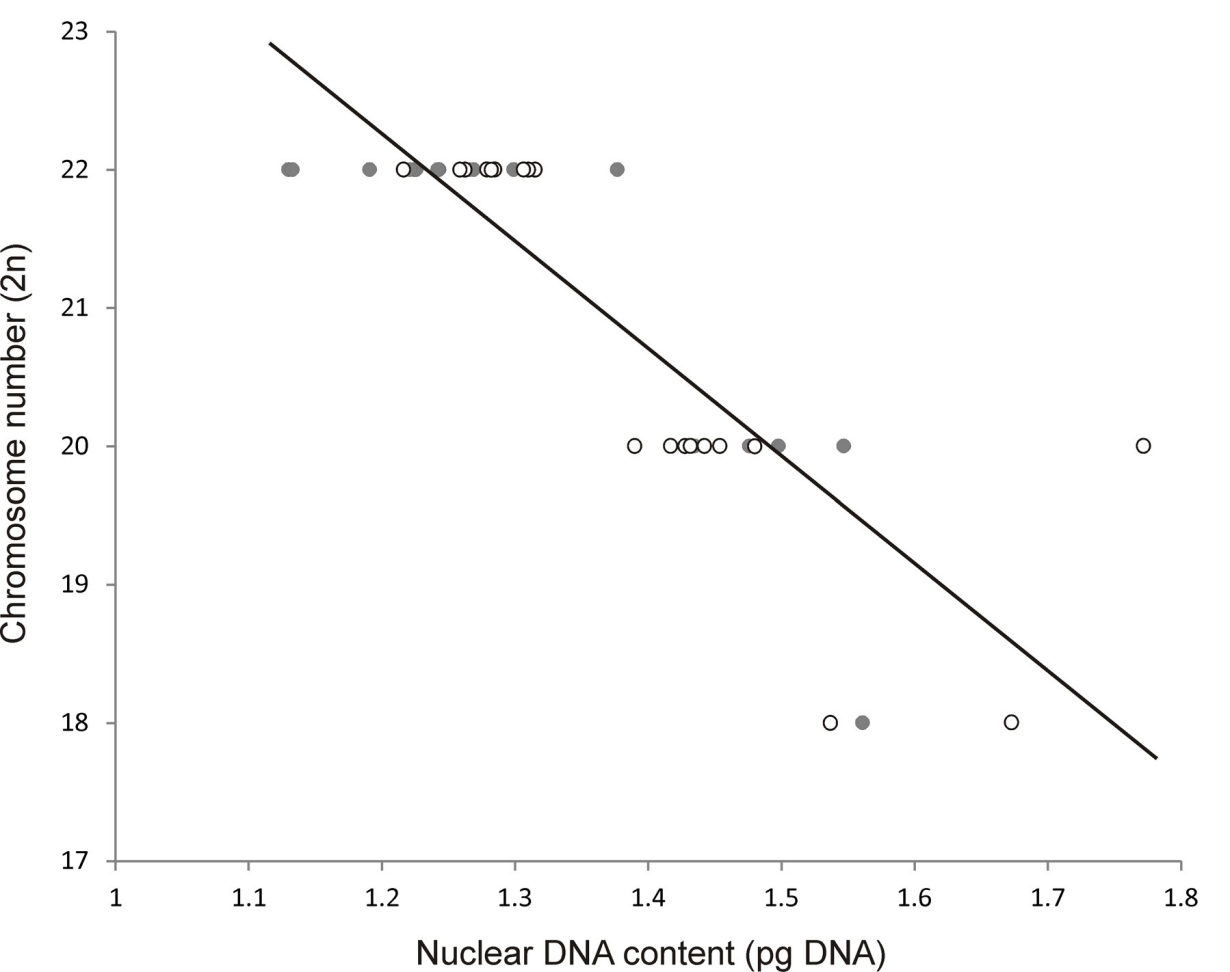
Relationship between nuclear 2C DNA content and 2n chromosome number in representatives of the genus *Musa* studied in the present work (empty circles) and by Bartoš *et al*. [9] (full circles), r = -0.85.

Spearman's correlation coefficient showed a strong negative correlation (r = -0.88) between 2n chromosome number and 2C nuclear DNA content among the *Musa* accessions. The same trend (r = -0.85) was observed when the results of Bartoš *et al*. [9] were included in the analysis (Fig 2). On the other hand, after including representatives of two other genera of the family Musaceae, *Ensete gilletii* [9] and *Musella lasiocarpa* (not published) the strong negative correlation between nuclear DNA content and chromosome number was no longer observed (r = -0.63).

### Chromosome Counting and Cytogenetic Mapping of rRNA Genes

Protoplast dropping technique was used to prepare metaphase spreads for chromosome counting and localization of rRNA genes. All accessions were confirmed to be diploid and their chromosome numbers corresponded to section-specific classification (Table 1). Representatives of the section Musa had 2n = 22 chromosomes, while Callimusa accessions had 2n = 18 (*M*. *beccarii*) or 2n = 20 chromosomes. Unfortunately, plants of *Musa exotica* (ITC.1532) and *M*. *campestris* var. *limbangensis* (ITC.1535) did not have actively growing roots needed for preparation of protoplast suspensions and their chromosomes could not be determined during this study.

**Fig 3:**
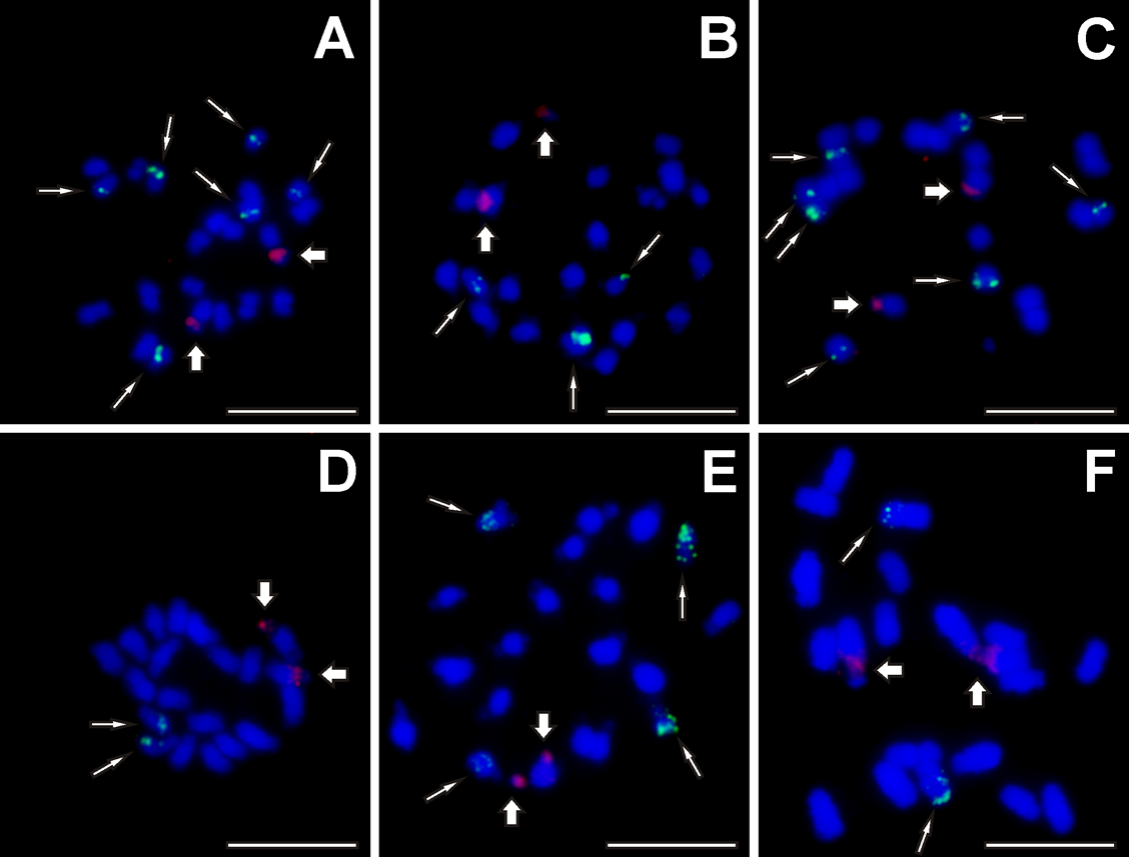
Examples of genomic distribution of 45S (red, thick arrows) and 5S (green, thin arrows) rRNA genes as localized on mitotic metaphase plates using FISH. (A) *Musa yunnanensis* (ITC.1573); (B) *Musa mannii* (ITC.1574); (C) *Musa rosea* x *siamensis* (ITC.1592); (D) *Musa campestris* var. *sarawakensis* (ITC.1517); (E) *Musa monticola* (ITC.1528); (F) *Musa lutea* (ITC.1515). Chromosomes were counterstained with DAPI. Bar = 5 μm.

FISH with probes for 45S rDNA revealed distinct hybridization sites on one chromosome pair in the secondary constriction in all accessions of the section Musa (Table 1, Fig 3). A variable number of 45S rDNA loci was observed in Callimusa species. Five accessions contained two signals (on one chromosome pair bearing secondary constriction), five signals were observed on chromosomes of *M*. *borneensis* (ITC.1531), while *M*. *beccarii* (ITC.1516 and ITC.1529) possessed six 45S rDNA loci on three chromosome pairs (Table 1). Two of these signals were observed in the secondary constriction of one chromosome pair, while the other sites of probe hybridization were localized in interstitial chromosome regions.

A significantly higher variation was detected in the number of 5S rDNA loci (Table 1). In a majority of accessions from the Musa section, 5S rDNA sites were localized on two or three chromosome pairs. Three signals were observed on chromosomes of *M*. *mannii* (ITC.1574, Fig 3B) and seven signals per metaphase plate were detected in *M*. *rosea* x *siamensis* (ITC.1592, Fig 3C). Callimusa species contained 5S rDNA gene clusters on two to eight chromosomes per metaphase plate. In *M*. *beccarii* var. *beccarii* (ITC.1516), *M*. *beccarii* var. *hottana* (ITC.1529) and *M*. *borneensis* (ITC.1531), two of the 5S rDNA sites co-localized with interstitially localized 45S rDNA genes.

### SSR Genotyping

**Fig 4:**
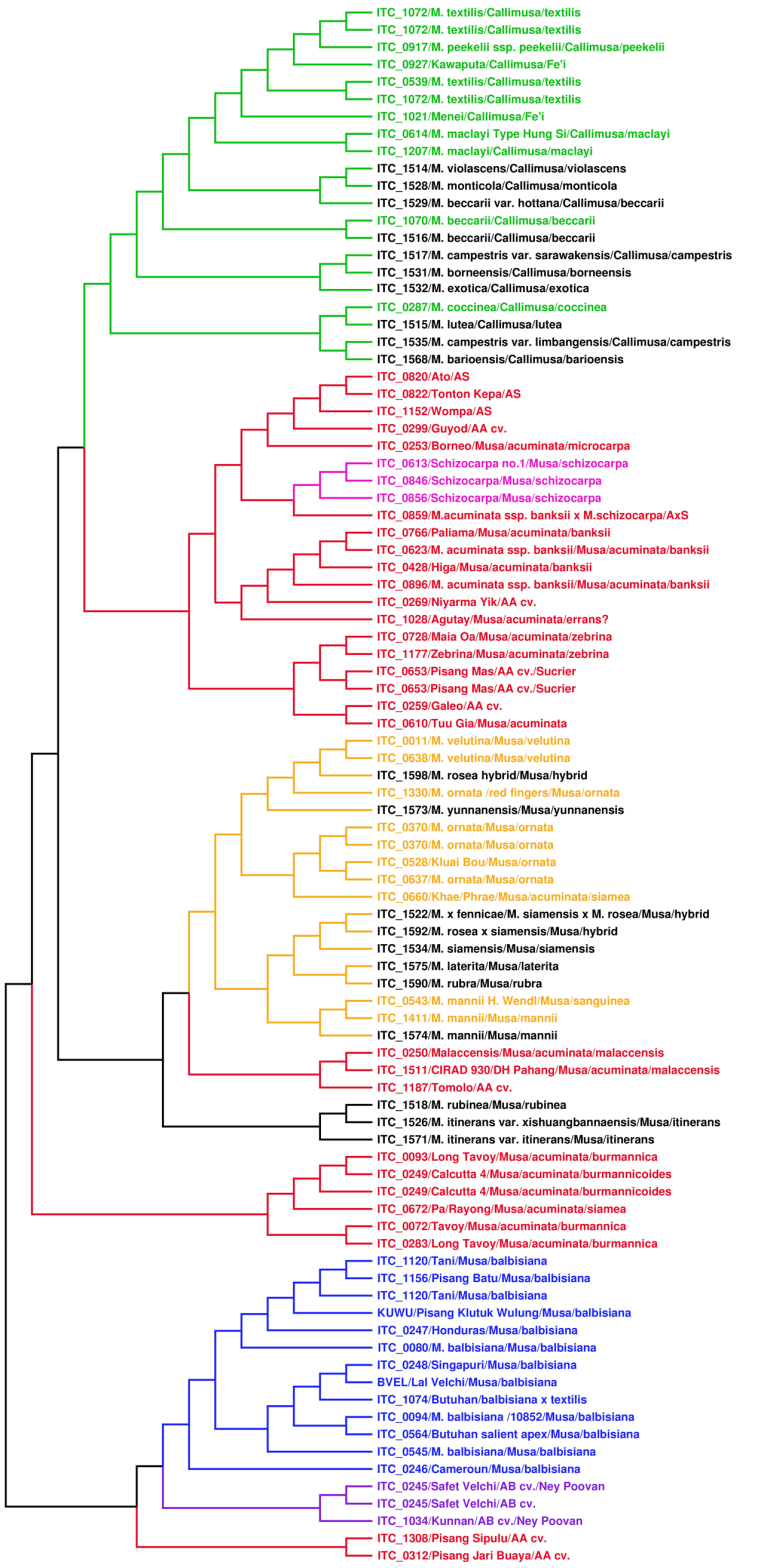
Dendrogram showing the results of the UPGMA analysis based on SSR markers. Accessions under study were analyzed together with the reference set of diploid entries as used by Christelová *et al*. [12]. The main clades and subclades are distinguished by colors. The Australimusa/Callimusa clade in green; Rhodochlamys in yellow; BB genotypes in blue; AA genotypes in red and SS genotypes in pink. Accessions analyzed in this study are shown in black.

Molecular characterization of the newly introduced ITC accessions based on SSR markers included inspection of their clustering pattern within the reference set of diploid entries used in the study of Christelová *et al*. [13]. The UPGMA cluster analysis based on the Nei [37] genetic distance resulted in relatively clear grouping of genotype groups and subgroups (Fig 4). Inclusion of the new accessions did not change the overall grouping of the species as compared to the results of Christelová *et al*. [13]. Most of the entries under this study clustered with the reference accessions as shown in our previous study [12] in accordance to their expected classification based on plant morphology. Newly introduced accessions belonging to the Musa section were grouped within the cluster A comprising other species from section Musa. The two accessions described as *M*. *itinerans* (ITC.1526 and ITC.1571) and *M*. *rubinea* (ITC.1518) formed a separate clade within the cluster A. Accessions described as Callimusa species were grouped within the cluster B together with reference accessions from this section. The allele sizes of analyzed accessions which were converted into binary code and added to the previous set of analyzed diploid *Musa* accessions [12] are available on the web pages of the *Musa* Genotyping Centre [31].

### Analysis of ITS1-5.8S-ITS2 Region

In general, the length of ITS1 and ITS2 spacer varied from 215 to 223 bp and from 205 to 218 bp, respectively. A total length of ITS1-5.8S-ITS2 sequence region ranged from 579 bp (*M*. *mannii*, ITC.1574) to 591 bp (*M*. *lutea*, ITC.1515) in all accessions, except for four representatives: *M*. *rubra* (ITC.1590), *M*. *rosea x siamensis* (ITC.1592), *M*. *yunnanensis* (ITC.1573) and *M*. *campestris* var. *sarawakensis* (ITC.1517) where the ITS region was shorter due to a deletion. The lowest nucleotide diversity of ITS1-5.8S-ITS2 sequence region was observed in *M*. *lutea* (ITC.1515) and *M*. *monticola* (ITC.1528). One of the highest sequence diversities were observed in accessions, which were described as wild diploid species: *M*. *mannii*, *M*. *yunnanensis*, *M*. *rubinea*, *M*. *siamensis*, *M*. *rubra*, *M*. *beccarii*, *M*. *borneensis*, *M*. *campestris* and *M*. *barionensis* (Table 2, S2 Table). GC content of ITS1 varied from 57.41 to 65.02% and was slightly lower than the GC content of ITS2 (59.51 to 69.74%). The 5.8S rDNA sequence region had a conserved length of 153 bp or 155 bp, except for two ITS types of *M*. *yunnanensis* (ITC.1573) and one type of *M*. *rubra* (ITC.1590) and M. *rosea* x *siamensis* (ITC.1598) due to the deletion. GC content of 5.8S rDNA varied between 46.45% and 59.63%, and was significantly lower than the GC content in ITS1 and ITS2.

**Table 2:** Sequence characteristics of ITS1-5.8S-ITS2 regions.

Accession name	ITS type	ITS1*	5.8S*	ITS2*	Motif M1**	Motif M2**	Motif M3**	Secondary structure of ITS2	Secondary structure of 5.8S	Note
*Musa rubinea*	1518_type1	57.67	53.55	63.76	conserved	conserved	conserved	four-helices	conserved	
	1518_type2	58.14	52.26	63.76	conserved	conserved	conserved	four-helices	conserved	
	1518_type3	62.96	57.42	69.55	conserved	conserved	conserved	four-helices	conserved	
*Musa x fennicae (M. siamensis (male) x M. rosea (female))*	1522_type1	62.50	57.42	69.63	conserved	conserved	conserved	four-helices	conserved	
	1522_type2	62.50	57.42	69.63	conserved	conserved	conserved	four-helices	conserved	
	1522_type3	61.57	57.42	69.16	conserved	conserved	conserved	four-helices	conserved	
	1522_type4	61.11	57.42	69.16	conserved	conserved	conserved	four-helices	conserved	
	1522_type5	62.50	57.42	69.16	conserved	conserved	conserved	four-helices	conserved	
*Musa itinerans var. xishuangbannaensis*	1526	57.67	53.55	63.76	conserved	conserved	conserved	four-helices	conserved	
*Musa siamensis*	1534_type1	58.80	52.26	62.74	nt-12 'A'	nt-9 'T'	conserved	four-helices	conserved	pseudogene
	1534_type2	57.41	46.45	62.15	nt-2 'A'	nt-6 'A'	nt-8 'A'	not formed	conserved	
						nt-9 'T'	nt-9 'T'			
						nt-14 'T'				
*Musa itinerans var. itinerans*	1571	57.67	53.55	63.76	conserved	conserved	conserved	four-helices	conserved	
*Musa yunnanensis*	1573_type1	63.59	57.42	68.84	conserved	conserved	conserved	four-helices	conserved	
	1573_type2	60.47	53.59	60.57	nt-2 'A'	conserved	conserved	not formed	not formed	pseudogene
	1573_type3	60.93	54.90	62.91	nt-2 'A'	conserved	conserved	four-helices	not formed	pseudogene
	1573_type4	57.41	48.94	64.02	nt-16 'T'	nt-9 'A'	nt-8 'A'	not formed	not formed	pseudogene
							nt-9 'T'			
*Musa mannii*	1574_type1	57.62	50.97	64.49	conserved	conserved	conserved	four-helices	conserved	
	1574_type2	58.72	53.55	68.22	conserved	conserved	conserved	four-helices	conserved	
	1574_type3	63.30	57.42	69.19	conserved	conserved	conserved	four-helices	conserved	
	1574_type4	57.80	52.26	63.55	conserved	nt-14 'T'	nt-8 'A'	not formed	conserved	pseudogene
*Musa laterita*	1575_type1	58.33	55.48	62.74	conserved	nt-9 'A'	conserved	four-helices	conserved	
	1575_type2	58.33	55.48	63.21	conserved	nt-9 'A'	conserved	four-helices	conserved	
*Musa rubra*	1590_type1	58.33	55.48	62.74	conserved	nt-9 'A'	conserved	four-helices	conserved	
	1590_type2	58.33	55.48	63.21	conserved	nt-9 'A'	conserved	four-helices	conserved	
	1590_type3	59.26	59.63	65.26	nt-11 'T'	not present-deletion	not present–deletion	not formed	not formed	pseudogene
*Musa rosea x siamensis*	1592_type1	61.29	57.42	68.69	conserved	conserved	conserved	four-helices	conserved	
	1592_type2	60.65	54.48	66.05	conserved	conserved	conserved	not formed	conserved	pseudogene
	1592_type3	58.80	55.86	63.51	conserved	nt1–nt3 'deletion' nt-9 'A'	conserved	four-helices	not formed	pseudogene
*Musa rosea (hybrid)*	1598	63.59	57.42	68.37	conserved	conserved	conserved	four-helices	conserved	
*Musa violascens*	1514	65.02	58.06	66.83	conserved	conserved	conserved	four-helices	conserved	
*Musa lutea*	1515	59.28	54.19	63.26	nt-11 'T'	conserved	nt-6 'T'	four-helices	conserved	
					nt-12 'A'					
*Musa beccarii var. beccarii*	1516_type1	65.77	58.06	65.85	conserved	conserved	conserved	four-helices	conserved	
	1516_type2	64.86	58.06	65.37	conserved	conserved	conserved	four-helices	conserved	
	1516_type3	65.32	58.06	65.20	conserved	conserved	conserved	four-helices	conserved	
*Musa campestris var. sarawakensis*	1517	59.64	48.95	61.46	nt-11 'T'	nt-7 'T'	conserved	four-helices	conserved	
					nt-12 'A'					
*Musa monticola*	1528	65.92	58.71	68.29	conserved	conserved	nt-3 'C'	four-helices	conserved	
*Musa beccarii var. hottana*	1529	65.32	58.06	65.37	conserved	conserved	conserved	four-helices	conserved	
*Musa borneensis*	1531_type1	65.02	57.42	65.37	conserved	conserved	conserved	four-helices	conserved	
	1531_type2	65.47	56.13	66.83	conserved	conserved	conserved	four-helices	conserved	
	1531_type3	65.92	56.77	68.29	conserved	conserved	conserved	four-helices	conserved	
	1531_type4	65.02	57.42	66.34	conserved	conserved	conserved	not formed	conserved	pseudogene
*Musa exotica*	1532_type1	63.68	58.06	66.83	conserved	conserved	conserved	four-helices	conserved	
	1532_type2	63.68	58.06	66.34	conserved	conserved	conserved	four-helices	conserved	
	1532_type3	65.02	58.06	66.83	conserved	conserved	conserved	four-helices	conserved	
*Musa campestris var. limbangensis*	1535_type1	64.13	58.06	65.85	conserved	conserved	conserved	four-helices	conserved	
	1535_type2	64.57	58.06	66.83	conserved	conserved	conserved	four-helices	conserved	
	1535_type3	56.50	50.32	59.51	nt-12 'A'	conserved	conserved	not formed	conserved	pseudogene
					nt-16 'A'					
*Musa barioensis*	1568_type1	61.88	56.13	62.80	nt-10 'G'	nt-9 'A'	nt-9 'T'	four-helices	conserved	
					nt-11 'A'					
	1568_type2	63.77	56.13	62.80	nt-10 'G'	nt-9 'A'	nt-9 'T'	four-helices	conserved	
					nt-11 'A'					
	1568_type3	65.92	58.06	68.29	conserved	conserved	conserved	four-helices	conserved	
	1568_type4	60.99	54.19	63.77	conserved	conserved	conserved	not formed	conserved	pseudogene

*) GC content [%]

**) Position of nucleotide changes (nt-) in conserved 5.8S motives

### Identification of rDNA Pseudogenes and Phylogenetic Analysis

The secondary structure of ITS2 and 5.8S rDNA sequence regions was reconstructed for all accessions under study. ITS2 sequences formed specific four-helices structure with typical pyrimidine-pyrimidine bulge in helix II and the most conserved primary sequence included the TGGT in the helix III. Secondary structure of 5.8S rDNA sequence was reconstructed under specific settings for base pairing as described by Hřibová *et al*. [33]. Moreover, 5.8S rDNA sequences were checked for the presence of three conserved motives [45]. The nucleotide changes in conserved motives of 5.8S rDNA of analyzed accessions, the information on GC content, presence of conserved motives in the 5.8S rDNA sequence and ability of ITS2 and 5.8S rDNA sequence to fold into a conserved secondary structure enabled identification of putative pseudogenes (Table 2). As shown in Table 2, more than half of the analyzed accessions contained at least two types of ITS sequences. On the contrary, *M*. *rosea* x *siamensis* ITC.1598 which has been described as hybrid clone had only one type of ITS sequence region.

With the aim to support clustering based on SSR markers, phylogenetic analysis of the ITS sequence region was done. Dataset for this analysis comprised ITS sequences of the *Musa* spp. previously described by Hřibová *et al*. [33] and ITS types of the 21 studied accessions, excluding the putative pseudogenic ITS types (S1 Fig). Inclusion of the new accessions did not change the overall grouping of the species as compared to the results of Hřibová *et al*. [33]. Phylogenetic analysis of ITS region supported the clustering obtained by SSR analysis for majority of the studied accessions. Close relationships of *M*. *itinerans* and *M*. *rubinea* accessions was supported by SSR as well as ITS analysis. On the other hand, ITS analysis indicated close relationships of these accessions to *M*. *balbisiana*, but this observation was not supported by the SSR analysis. Similarly, close relationships of *M*. *yunnanensis* (ITC.1573) and *M*. *rosea* hybrid (ITC.1598) to *M*. *ornata* and *M*. *velutina* obtained after SSR analysis was not supported by ITS analysis. Finally, different clustering was observed for accession *Musa x fennicae (Musa siamensis (male) x Musa rosea (female)* (ITC.1522) which is found in the cluster containing ornamental bananas (former Rhodochlamys section) in the SSR cladogram while using ITS analysis, all ITS sequence types including putative pseudogenes are clustered together with AA genotypes.

In our previous study [33], we have shown that, with certain caution, pseudogenic rDNA sequences can be used to identify parental genotypes of hybrid clones. Considering the fact that more than a half of the analyzed accessions contained at least two types of ITS sequences, phylogenetic reconstruction with ITS sequences including putative pseudogenes was done (S2 Fig). As it is visible from the ITS clustering, the genome of *Musa yunnanensis* (ITC.1573) contains divergent ITS sequence types which clustered together with ornamental bananas as well as with AA genotypes. Similar observation was obtained also for genotype described as hybrid clones of ornamental banana genotypes—*M*. *rosea* x *siamensis* (ITC.1592), while hybrid clone *Musa x fennicae (Musa siamensis (male) x Musa rosea (female)* (ITC.1522) contained only ITS sequence types which clustered together with the AA genotypes (see above).

## Discussion

### Nuclear DNA Content and Distribution of rDNA

Most of the knowledge on nuclear genome size and genomic distribution of rDNA loci in banana comes from the analysis of triploid cultivars and their wild ancestors [14–16, 17, 18, 46]. Only the study of Bartoš *et al*. [9] included a wider range of *Musa* species and provided the first complex picture of the whole genus. Our study significantly expands the number of wild *Musa* species where these key characteristics of nuclear genome are described.

The diversity in genome size observed in species belonging to the section Musa is in line with previous studies [9, 14–16, 46] which estimated 2C nuclear DNA content ranging from 1.108 pg in *M*. *balbisiana* to 1.377 pg in *M*. *schizocarpa*. The genome size of *M*. *laterita* and *M*. *mannii* were determined by Bartoš *et al*. [9]. However, in both cases, different ITC accessions (ITC.0627 and ITC.1411, respectively) were used. While the difference in 2C nuclear DNA content estimates between both accessions (ITC.1411 and ITC.1574) of *M*. *mannii* is negligible (1.0%), there is much higher difference (7.7%) between *M*. *laterita* accessions (ITC.0627 and ITC.1575). This observation suggests that accessions ITC.0627 and ITC.1575 could represent different varieties of *M*. *laterita*.

Genomic distribution of 45S and 5S rDNA clusters in *Musa* accessions as observed in this study is similar to the results obtained by Bartoš *et al*. [9] and together with overlapping genome size values brings further evidence about the close relationships between representatives of former sections Eumusa and Rhodochlamys. The traditional division into sections Eumusa and Rhodochlamys was mainly based on morphological characters. The separation of these sections was however doubtful, because morphological differences were difficult to detect in some species and interspecific hybridization was frequent [6, 47]. Our findings, as well as previous analyses based on different molecular markers [8, 10, 21, 23], support the recent merger of sections Eumusa and Rhodochlamys into the section Musa.

The presence of odd numbers of 5S rDNA loci has been previously observed in some edible cultivars and wild species [9, 17] and could be explained by structural chromosome heterozygosity and/or as a result of hybridization between two genotypes bearing different 5S rDNA sites. However, it is also possible that one of 5S rDNA loci contained a lower number of repeat units and the resulting signal was below the detection limit of FISH.

The representatives of the section Callimusa showed high variation in nuclear genome size as well as in the number of rDNA loci and were clearly discriminated from the accessions of the section Musa. For *M*. *borneensis* (ITC.1531), the highest nuclear DNA content (2C = 1.772 pg) known for diploid *Musa* species was determined. The increased genome size was accompanied by a higher number of 45S and 5S rDNA loci in comparison with other Callimusa accessions. Until now, the largest genome size was detected in *M*. *beccarii* [9, 48], which has also been characterized by more abundant distribution of rRNA genes. The present estimates of genome sizes as well as chromosome number and distribution of rDNA loci in both *M*. *beccarii* varieties are in line with previous investigations [9, 48].

An interesting outcome of this study is the observation of a negative correlation between the basic chromosome number (x) and nuclear genome size (1C), which became evident in this study after a wider range of *Musa* species was analyzed. This trend could be explained by the evolution from a common ancestor by chromosome fission accompanied by DNA loss. This hypothesis is in line with Simmonds [49], who speculated that the lower chromosome number of *M*. *beccarii* and the genus *Ensete* reflects their ancient origin. Similarly, Bekele and Shigeta [50] suggested that *Ensete glaucum* and *M*. *beccarii* are ancestral forms of *Ensete* and *Musa* with a common ancestor that is yet to be established and the ancestral chromosomal number of both genera is x = 9. On the other hand, if this hypothesis was true, representatives of the genera *Ensete* and *Musella* would be expected to have a genome size comparable to that of and other Callimusa species. A more extensive study of the karyotype evolution in the family Musaceae will be necessary to explain this "the less chromosomes, the larger genome" trend emerging in the genus *Musa*, but not observed in other genera of the family.

### SSR and ITS Analysis

SSR analysis provided important information about the genomes of 21 accessions of wild *Musa* species, which were genotyped using molecular markers for the first time. The grouping of the accessions within the diploid reference set was revealed by the UPGMA cluster analysis. In most cases, the results of this grouping (Fig 3) were consistent with the characterization based on traditional morpho-taxonomic classification. Apart from better characterization of the wild *Musa* species, the SSR profiles obtained in this work expand the database of reference SSR profiles and thus improves the grouping and enhances the probability of identifying the closest relative or an exact match for unknown accessions.

Short length, utilization of highly conserved primers and relatively fast evolution of internal transcribed spacers ITS1 and ITS2 as compared to rRNA genes, made the ITS region one of the most popular markers in phylogenetic studies. The first detailed information about the structure and diversity of ITS region in the genus *Musa* was provided by Hřibová *et al*. [33], while the work of Christelová *et al*. [12] showed the usefulness of the ITS for further analysis of accessions which give incongruous results with SSRs. High level of nucleotide diversity and presence of more than two types of ITS1-5.8S-ITS2 sequence region in eight wild diploid accessions (*M*. *yunnanesis*, *M*. *rubinea*, *M*. *rubra*, *M*. *exotica*, *M*. *beccarii* var. *beccarii*, *M*. *borneensis*, *M*. *mannii*, *M*. *barionensis*) indicate that they have originated from hybridization of different genotypes.

The close relationship of *M*. *itinerans* to B genome representatives observed after ITS analysis is in agreement with the study of Liu *et al*. [11]. This relationship was not supported by the SSR analysis. It is, however, possible that the grouping of some species will change after more representatives from both sections pass through the SSR genotyping pipeline. In a similar way, we have observed discrepancies in the position of *M*. *yunnanensis* (ITC.1573) and *M*. *rosea* hybrid (ITC.1598), as well as *Musa x fennicae (Musa siamensis (male) x Musa rosea (female)* (ITC.1522) in the tree constructed from SSR and ITS data (see above; Fig 4, and S1 and S2 Figs). The ITS analysis indicates that the *M*. *yunnanensis* accessions included in our study originated from hybridization of different genotypes (subspecies?) (Table 2), while the presence of only one ITS type sequence in the accession labeled as *M*. *rosea* hybrid (ITC.1598) does not support its hybrid character. In *Musa x fennicae (Musa siamensis (male) x Musa rosea (female)* (ITC.1522), divergent types of ITS sequences were identified but all of them were related to AA genotypes, while SSR analysis showed close relationships of this clone with ornamental bananas (genotypes of former Rhodochlamys section). These observed discrepancies could be a consequence of dominance of one type of rDNA sequence in hybrids, which may lead to complete homogenization of rDNA locus originating from the second parent [51–53].

Our results confirm the usefulness of SSR markers for molecular characterization of unknown accessions of *Musa* [12]. As the SSR genotyping is based on scoring alleles, it may not be appropriate for diversity and phylogenetic inference estimation in case of inter-specific hybrids and their backcross progenies [33]. Thus, we performed a detailed analysis of the nucleotide composition and structure of the ITS region and showed that some of the wild diploids contained polymorphic ITS regions. Moreover, *in silico* analysis of the ITS sequences indicated the presence of putative pseudogenic ITS types. These observations indicate that some of the analyzed accessions of wild *Musa* species originated from hybridization between different genotypes within a species or between putative subspecies [33, 54].

## Conclusions

This study improves significantly the knowledge about the nuclear genome of wild *Musa* species. We provide novel data on nuclear genome size and genomic distribution of ribosomal genes in 21 wild *Musa* accessions. We revealed high variability in both characters, especially in the section Callimusa. Our results indicate that species of the *Musa* genus evolved from a common ancestor by chromosome fission, which was accompanied by DNA loss. Cladogram based on the SSR markers together with the previously studied wild diploids showed clear clustering and significantly increased the knowledge on genetic diversity within *Musa* genus. SSR genotyping platform enabled us to identify groups of the most related species and sub-species. Apart from phylogenetic analysis, characterization of ITS sequences indicated that more than a half of the new ITC accessions representing wild *Musa* accessions originated from hybridization between different genotypes within a species or between putative subspecies.

## Supporting Information

S1 Fig Phylogenetic analysis based on the ITS1-ITS2 sequence region according to Hřibová *et al*. [34]. BioNJ tree constructed from a Jukes-Cantor distance matrix of the concatenated region containing ITS1 and ITS2 spacer sequence. Closely related species *Strelitzia* and *Heliconia* were used as outgoup. The tree was rooted on midpoint. The main clades and subclades are distinguished by colors. The Australimusa/Callimusa clade in green; Rhodochlamys in yellow; BB genotypes in blue; AA genotypes in red and SS genotypes in pink. The ITS sequences of accessions analyzed in this study are shown in black.
(TIF)

S2 Fig Phylogenetic analysis based on the ITS1-ITS2 sequence region according to Hřibová *et al*. [34]. BioNJ tree constructed from a Jukes-Cantor distance matrix of the concatenated region containing ITS1 and ITS2 spacer sequence including putative pseudogenic sequences. Closely related species *Strelitzia* and *Heliconia* were used as outgoup. The tree was rooted on midpoint. The main clades and subclades are distinguished by colors. The Australimusa/Callimusa clade in green; Rhodochlamys in yellow; BB genotypes in blue; AA genotypes in red and SS genotypes in pink. The ITS sequences of analyzed accessions are in black color. Putative pseudogenic ITS sequence regions are marked with asterisk.
(TIF)

S1 Table The origin of *Musa* accessions analyzed in this study. (DOCX)

S2 Table Nucleotide diversity of ITS1-5.8S-ITS2 regions. (DOCX)
