# Supplementary figures and images for: Molecular and Cytogenetic Characterization of Wild Musa Species
Source: PLoS One. 2015 Aug 7;10(8):e0134096. doi: 10.1371/journal.pone.0134096 (PMC4529165; doi:10.1371/journal.pone.0134096)

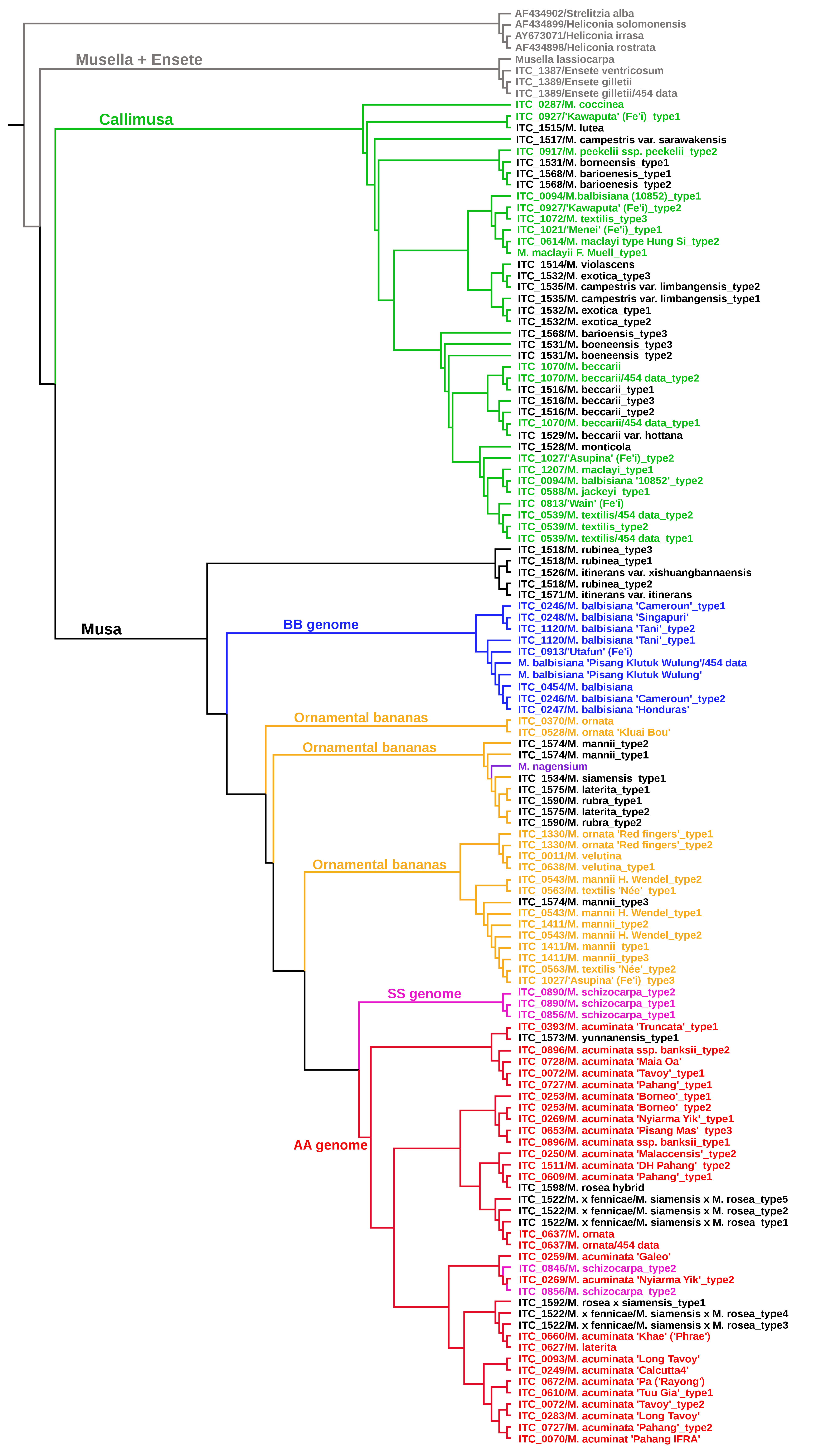

Supplement: S1 Fig — BioNJ tree constructed from a Jukes-Cantor distance matrix of the concatenated region containing ITS1 and ITS2 spacer sequence. Closely related species Strelitzia and Heliconia were used as outgoup. The tree was rooted on midpoint. The main clades and subclades are distinguished by colors. The Australimusa/Callimusa clade in green; Rhodochlamys in yellow; BB genotypes in blue; AA genotypes in red and SS genotypes in pink. The ITS sequences of accessions analyzed in this study are shown in black. (TIF) [file pone.0134096.s001.tif]

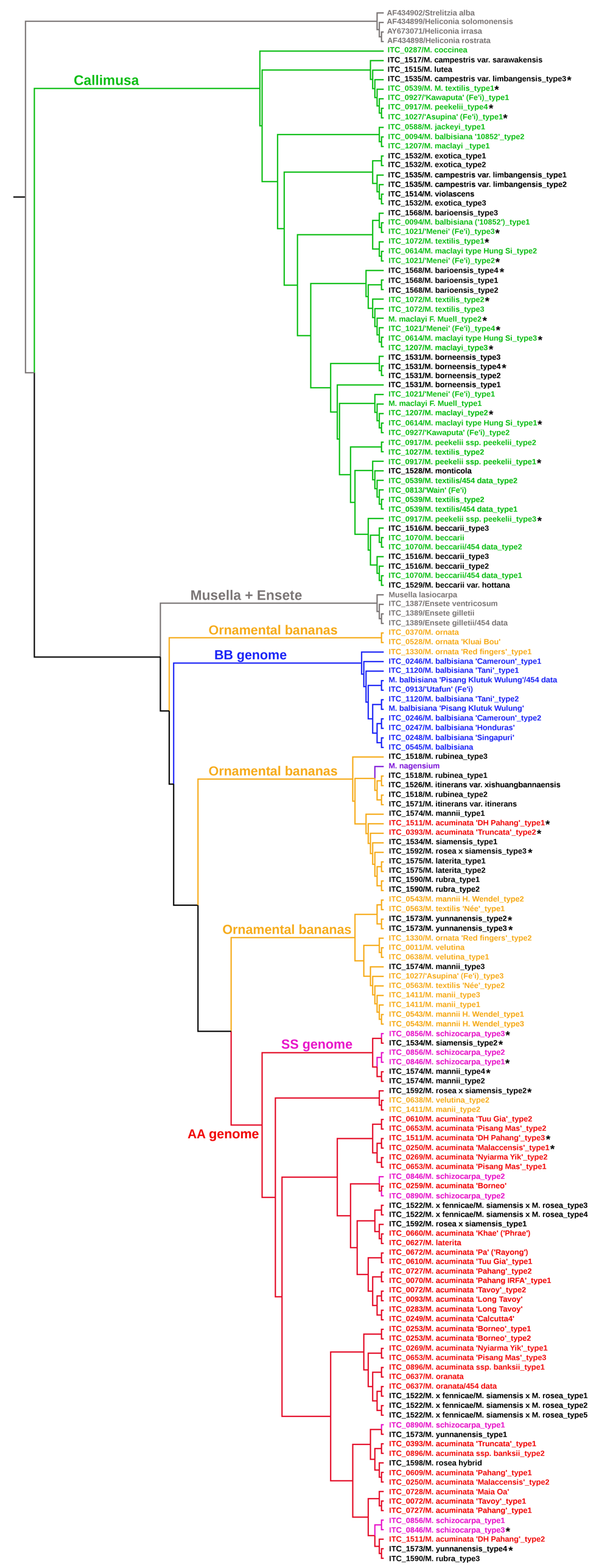

Supplement: S2 Fig — BioNJ tree constructed from a Jukes-Cantor distance matrix of the concatenated region containing ITS1 and ITS2 spacer sequence including putative pseudogenic sequences. Closely related species Strelitzia and Heliconia were used as outgoup. The tree was rooted on midpoint. The main clades and subclades are distinguished by colors. The Australimusa/Callimusa clade in green; Rhodochlamys in yellow; BB genotypes in blue; AA genotypes in red and SS genotypes in pink. The ITS sequences of analyzed accessions are in black color. Putative pseudogenic ITS sequence regions are marked with asterisk. (TIF) [file pone.0134096.s002.tif]
