# Supplementary material for: Molecular and Cytogenetic Characterization of Wild Musa Species
Source: PLoS One. 2015 Aug 7;10(8):e0134096. doi: 10.1371/journal.pone.0134096 (PMC4529165; doi:10.1371/journal.pone.0134096)
Supplement: S1 Table — (DOCX) [file pone.0134096.s003.docx]

**S1 Table.** The origin of *Musa* accessions analyzed in this study.

| **Accession name** | **ITC code*** | **Donated to ITC by** | **Original collection site** | **Description (reference)** |
| --- | --- | --- | --- | --- |
| *Musa rubinea* | 1518 | Helsinki University Botanic Garden | Western Yunnan, China | [55] |
| *Musa x fennicae* | 1522 | Helsinki University Botanic Garden | Finnland | Not published |
| *Musa itinerans* var. *xishuangbannaensis* | 1526 | Helsinki University Botanic Garden | Yunnan, China | [56] |
| *Musa siamensis* | 1534 | Helsinki University Botanic Garden | Thailand | [57] |
| *Musa itinerans* var. *itinerans* | 1571 | Helsinki University Botanic Garden | Yunnan, China | [56] |
| *Musa yunnanensis* | 1573 | Helsinki University Botanic Garden | Yunnan, China | [58] |
| *Musa mannii* | 1574 | Helsinki University Botanic Garden | Tenerife, Canary Islands | [59] |
| *Musa laterita* | 1575 | Helsinki University Botanic Garden | India | [60] |
| *Musa rubra* | 1590 | Helsinki University Botanic Garden | Northeast India | [61, 62] |
| *Musa rosea* x *siamensis* | 1592 | Helsinki University Botanic Garden | Finnland | Not published |
| *Musa rosea* (hybrid) | 1598 | Helsinki University Botanic Garden | Finnland | Not published |
| *Musa violascens* | 1514 | Helsinki University Botanic Garden | Peninsular Malaysia | [63] |
| *Musa lutea* | 1515 | Helsinki University Botanic Garden | North Vietnam | [14, 64] |
| *Musa beccarii* var. *beccarii* | 1516 | Helsinki University Botanic Garden | Sabah, Borneo | [48] |
| *Musa campestris* var. *sarawakensis* | 1517 | Helsinki University Botanic Garden | Sarawak, Borneo | [65, 66] |
| *Musa monticola* | 1528 | Helsinki University Botanic Garden | Sabah, Borneo | [67] |
| *Musa beccarii* var. *hottana* | 1529 | Helsinki University Botanic Garden | Sabah, Borneo | [48] |
| *Musa borneensis* | 1531 | Helsinki University Botanic Garden | Sarawak, Borneo | [68] |
| *Musa* *exotica* | 1532 | Helsinki University Botanic Garden | Vietnam | [69] |
| *Musa campestris* var. *limbangensis* | 1535 | Helsinki University Botanic Garden | Sarawak, Borneo | [65, 66] |
| *Musa barioensis* | 1568 | Helsinki University Botanic Garden | Sabah, Borneo | [70] |

*) International *Musa* Germplasm Transit Centre

**References**

55. **Häkkinen M, Teo CH.** *Musa rubinea*, a new *Musa* species (Musaceae) from Yunnan, China. Folia Malaysiana. **2008; 9(1)**: 23-33.

56. **Häkkinen M, Hong W, Ge X-J.** *Musa itinerans* (Musaceae) and its intraspecific taxa in Chi-na. Novon. **2008; 18(1)**: 50-60.

57. **Häkkinen M, Wallace R.** *Musa siamensis*, a new species (Musaceae) from SE Asia. Folia Malaysiana. **2007; 8(2)**: 61-70.

58. **Häkkinen M, Wang H.** New species and variety of *Musa* (Musaceae) from Yunnan, China. Novon. **2007; 17(4)**: 440-446.

59. **Häkkinen M, Väre H.**Typification of *Musa mannii*, *M. sanguinea* and *M. x kewensis* (Musaceae). Kew Bulletin. **2009; 64(3)**: 559-564.

60. **Häkkinen M.** *Musa laterita*: An ornamental banana. Fruit Gardener. **2001; 33(4)**: 6-7.

61. **Häkkinen M.** Taxonomic history and identity of *Musa rubra* Wall. ex Kurz. Philipp Agric Sci. **2003; 86(1)**: 92-98.

62. **Häkkinen M.** *Musa chunii* Häkkinen, a new species (Musaceae) from Yunnan, China and taxonomic identity of *Musa rubra*. J Syst Evol. **2009; 47(1)**: 87-91.

63. **Ridley HN.** *Musa violascens*. Trans Linn Soc Ser 2. **1893; 3**: 384.

64. **Valmayor RV.** Classification and characterization of *Musa exotica*, *M. alinsanaya* and *M. acuminata* ssp. *errans*. Philipp Agric Sci. **2001; 84(3)**: 325-331.

65. **Häkkinen M.** *Musa campestris* Beccari varieties in northern Borneo. Philipp Agric Sci. **2003; 86(4)**: 424-435.

66. **Häkkinen M.** *Musa campestris* Becc. (Musaceae) varieties in northern Borneo. Folia Malaysiana. **2004; 5(2)**: 81-100.

67. **Argent GCG.** Two interesting wild *Musa* species (Musaceae) from Sabah, Malaysia. Gardens' Bulletin - Singapore. **2000; 52**: 203-210.

68. **Häkkinen M, Meekiong K.** *Musa borneensis* Becc. (Musaceae) and its intraspecific taxa in Borneo. Acta Phytotax Geobot. **2005; 56(3)**: 213-230.

69. **Valmayor RV, Danh L, Häkkinen M.** Rediscovery of *Musa splendida* A. Chevalier and de-scription of two new species (*Musa viridis* and *Musa lutea*). Philipp Agric Sci. **2004; 87(1)**: 110-18.

70. **Häkkinen M.** *Musa barioensis*, a new *Musa* species (Musaceae) from northern Borneo. Acta Phytotax Geobot. **2006; 57(1)**: 55-60.
