# Supplementary material for: Molecular and Cytogenetic Characterization of Wild Musa Species
Source: PLoS One. 2015 Aug 7;10(8):e0134096. doi: 10.1371/journal.pone.0134096 (PMC4529165; doi:10.1371/journal.pone.0134096)
Supplement: S2 Table — (DOCX) [file pone.0134096.s004.docx]

**S2 Table.** Nucleotide diversity of ITS1-5.8S-ITS2 regions.

| **Section of the *Musa* genus** | **Accession name** | **ITC code*** | **No. of sequenced clones** | **Nucleotide diversity θπ** |
| --- | --- | --- | --- | --- |
| Musa | *Musa rubinea* | 1518 | 30 | 24.021 |
|  | *Musa x fennicae* (*M. siamensis* (male) x *M. rosea* (female)) | 1522 | 75 | 8.155 |
|  | *Musa itinerans* var. *xishuangbannaensis* | 1526 | 31 | 9.202 |
|  | *Musa siamensis* | 1534 | 30 | 25.644 |
|  | *Musa itinerans* var. *itinerans* | 1571 | 30 | 9.202 |
|  | *Musa yunnanensis* | 1573 | 46 | 27.513 |
|  | *Musa mannii* | 1574 | 60 | 36.304 |
|  | *Musa laterita* | 1575 | 32 | 5.452 |
|  | *Musa rubra* | 1590 | 62 | 15.528 |
|  | *Musa rosea* x *siamensis* | 1592 | 91 | 13.266 |
|  | *Musa rosea* (hybrid) | 1598 | 67 | 7.303 |
| Callimusa | *Musa violascens* | 1514 | 37 | 3.667 |
|  | *Musa lutea* | 1515 | 28 | 0.668 |
|  | *Musa beccarii* var. *beccarii* | 1516 | 58 | 11.574 |
|  | *Musa campestris* var. *sarawakensis* | 1517 | 32 | 2.054 |
|  | *Musa monticola* | 1528 | 37 | 0.963 |
|  | *Musa beccarii* var. *hottana* | 1529 | 32 | 12.695 |
|  | *Musa borneensis* | 1531 | 54 | 14.894 |
|  | *Musa* *exotica* | 1532 | 75 | 5.214 |
|  | *Musa campestris* var. *limbangensis* | 1535 | 42 | 17.336 |
|  | *Musa barioensis* | 1568 | 55 | 20.506 |

*) International *Musa* Germplasm Transit Centre
